# Editorial: Physical education, health and education innovation

**DOI:** 10.3389/fpsyg.2024.1458407

**Published:** 2024-07-24

**Authors:** David Manzano-Sánchez, Noelia Belando-Pedreño, Jorge Carlos-Vivas, Paulo Jorge Martins, Manuel Gómez-López

**Affiliations:** ^1^Faculty of Education and Psychology, University of Extremadura, Badajoz, Spain; ^2^Faculty of Sport Sciences, European University of Madrid, Villaviciosa de Odón, Spain; ^3^Faculty of Human Motricity, University of Lisbon, Dafundo, Portugal; ^4^Faculty of Sports Sciences, University of Murcia, Murcia, Spain

**Keywords:** physical activity, high school, children, pedagogical models, motor competence, health

Physical Education is closely linked with the overall health of individuals, with education, and with educational innovation essential aspects for shaping the development of individuals within the educational context. Thus, these aspects form part of the educational process, which encompasses knowledge from applied psychology to education. Examples include the analysis of motivation in the teaching-learning process, the analysis of physical exercise behavior, and adherence to sports practice at early ages. In this context, various studies highlight the importance of interventions among children and adolescents to promote physical fitness due to its relationship with adequate physical aptitude, motor skills, and overall wellbeing in later stages (young adulthood, adulthood, and even older adulthood). For instance, in the meta-analysis by Li H. et al., it was observed that physical fitness and basic motor skills interventions improved these capacities and skills with sessions lasting 60 min, practiced 1–3 days a week for at least 16 weeks. Along the same lines, Chen J. et al. report on the importance of interventions through movement during preschool stages. These authors conclude that physical fitness and fundamental motor skills mutually enhance each other in young children, and both should be emphasized in preschool sports education. Similarly, the development of motor literacy can be crucial for engaging in physical activity over the years, finding that individuals with higher motor competence across various dimensions also show higher levels of moderate to vigorous physical activity (Martinez-Lopez et al.). Thus, there is a need to promote the development of motor competence to increase the rate of physical activity and sports activities among schoolchildren.

Regarding adolescence, the educational context could be decisive in the levels of physical exercise practice and its impact on normative behavior, as suggested by Chen H. et al.'s study where the use of the STEAM teaching method improved engagement with learning. Additionally, the study by Rojo-Ramos et al. found that in adolescence and pre-adolescence, higher self-efficacy is associated with lower levels of abuse and victimization, positioning regular physical activity as a mediator for preventing cyberbullying.

In relation of the concept of innovation, responsible and proportionate use of virtual reality could foster a positive attitude among basketball players and achieve professional success in the classroom, as indicated by Wang. Other studies, such as Guijarro-Romero et al., evaluate the use of technologies, for example, the inclusion of activity bracelets and behavior modification techniques in training activities. These authors found highly satisfactory results, as they support perceived autonomy and increase physical activity levels among adolescents. Similarly, Chow and Mann shows that ‘exergaming' or games promoting physical activity through technology could contribute to improving healthy habits. Their study is based on a theoretical model using Bloom's taxonomy to obtain resources for future research on exergaming.

In the field of Physical Education, small-sided games allow high-intensity physical activity in classes flexibly and motivatingly, with many possibilities for class design and application across different sports (Li Q. Z. et al.). Thus, educational innovation is necessary within Physical Education through model hybridization, as evidenced by Quiñonero-Martínez et al. study, with positive results toward creating habits related to physical activity. Similarly, the use of alternative sports, as shown in Diez-Fernández et al. study with the practice of “cornerball,” is an appropriate alternative for promoting sports in different ways. Teaching artistic activities and not just sports can also greatly benefit students by improving aspects such as self-efficacy and self-esteem in young students (Zhou et al.). Moreover, scientific literature indicates that achieving higher academic performance necessitates appropriate physical activity, finding that students who run at least once a week excel academically with a sample of over 2200 university participants (Du et al.).

Finally, innovation should be present not only in Physical Education classes but also in the sports field, developing intervention program like the Real Madrid Foundation (RMF) by Ortega-Vila et al., which show conclusive results toward personal and team success, self-fulfillment, personal and group superiority, health, and physical fitness.

Another essential aspect of the educational process, along with psychology, physical exercise behavior, and innovation, is teacher training to integrate all these concepts for the overall health development of schoolchildren and adolescents. Hence, developing theoretical models to improve the communication skills of teachers and physical trainers and providing them with didactic resources to foster learning climates based on more self-determined motivation is important (Chen L. et al.). Since motivation drives human behavior, it is necessary to understand how to internally motivate children and adolescents, especially from early ages, to foster values such as personal and social responsibility and the intention to engage in physical activity in the present and future (Manzano-Sánchez). Closely linked to motivation, emotional intelligence could influence increased life satisfaction and reduced anxiety levels, making its study essential. It seems that clarity and emotional repair variables can act as mediators, reduces the negative effects of anxiety (Calleja-Núñez et al.). Other psychological aspects that explain human behavior, such as personality traits, are fundamental in Physical Education classes, as they can predict satisfaction with classes, as indicated by Chen Z. et al. in older students.

Finally, it is important to consider the age and sex of students, as these aspects can influence the degree of physical activity, making it advisable to implement study plans that address these differences in Physical Education classes, investing in appropriate facilities and materials (Ma et al.).

## Author contributions

DM-S: Conceptualization, Writing – original draft, Writing – review & editing. NB-P: Writing – original draft, Writing – review & editing, Conceptualization. JC-V: Supervision, Validation, Writing – review & editing. PM: Supervision, Validation, Writing – review & editing. MG-L: Supervision, Validation, Writing – review & editing.

